# Antimicrobial resistance predicts death in Tanzanian children with bloodstream infections: a prospective cohort study

**DOI:** 10.1186/1471-2334-7-43

**Published:** 2007-05-22

**Authors:** Bjørn Blomberg, Karim P Manji, Willy K Urassa, Bushir S Tamim, Davis SM Mwakagile, Roland Jureen, Viola Msangi, Marit G Tellevik, Mona Holberg-Petersen, Stig Harthug, Samwel Y Maselle, Nina Langeland

**Affiliations:** 1 Department of Medicine, Haukeland University Hospital, Bergen, Norway; 2 Institute of Medicine, University of Bergen, Norway; 3 Centre for International Health, University of Bergen, Norway; 4 Department of Paediatrics and Child Health, Muhimbili University College of Health Sciences, Dar es Salaam, Tanzania; 5 Department of Microbiology and Immunology, Muhimbili University College of Health Sciences, Dar es Salaam, Tanzania; 6 Department of Laboratory Medicine, Alexandra Hospital, Singapore; 7 Department of Microbiology, Ullevål University Hospital, Oslo, Norway; 8 Faculty of Medicine, University of Oslo, Norway

## Abstract

**Background:**

Bloodstream infection is a common cause of hospitalization, morbidity and death in children. The impact of antimicrobial resistance and HIV infection on outcome is not firmly established.

**Methods:**

We assessed the incidence of bloodstream infection and risk factors for fatal outcome in a prospective cohort study of 1828 consecutive admissions of children aged zero to seven years with signs of systemic infection. Blood was obtained for culture, malaria microscopy, HIV antibody test and, when necessary, HIV PCR. We recorded data on clinical features, underlying diseases, antimicrobial drug use and patients' outcome.

**Results:**

The incidence of laboratory-confirmed bloodstream infection was 13.9% (255/1828) of admissions, despite two thirds of the study population having received antimicrobial therapy prior to blood culture. The most frequent isolates were klebsiella, salmonellae, *Escherichia coli*, enterococci and *Staphylococcus aureus*. Furthermore, 21.6% had malaria and 16.8% HIV infection. One third (34.9%) of the children with laboratory-confirmed bloodstream infection died. The mortality rate from Gram-negative bloodstream infection (43.5%) was more than double that of malaria (20.2%) and Gram-positive bloodstream infection (16.7%). Significant risk factors for death by logistic regression modeling were inappropriate treatment due to antimicrobial resistance, HIV infection, other underlying infectious diseases, malnutrition and bloodstream infection caused by *Enterobacteriaceae*, other Gram-negatives and candida.

**Conclusion:**

Bloodstream infection was less common than malaria, but caused more deaths. The frequent use of antimicrobials prior to blood culture may have hampered the detection of organisms susceptible to commonly used antimicrobials, including pneumococci, and thus the study probably underestimates the incidence of bloodstream infection. The finding that antimicrobial resistance, HIV-infection and malnutrition predict fatal outcome calls for renewed efforts to curb the further emergence of resistance, improve HIV care and nutrition for children.

## Background

One in every six African children dies before the age of five years [1]. The World Health Organization (WHO) rank the major causes of mortality in African children younger than five years as neonatal causes (26%, among which the entity "sepsis or pneumonia" contributes a quarter), pneumonia (21%), malaria (18%) diarrhea (16%) and HIV-infection (6%) [2]. Bloodstream infection is a frequent cause of morbidity and associated with mortality in excess of 25% [3]. Since bloodstream infection may occur as part of localized infections with defined foci such as pneumonia and diarrhea, its importance is not reflected in the above estimates of death causes. Bloodstream infection and malaria are practically indistinguishable by clinical examination [4], and available WHO guidelines for managing childhood illnesses fail to identify up to half of the cases of bloodstream infections [5]. A recent study from Kenya [3] found that bloodstream infection caused one quarter of all deaths of children in the hospital, outnumbering malaria deaths. Antimicrobial resistance increases worldwide and does not spare developing countries [6]. However, the impact of antimicrobial resistance on the clinical outcome of infections such as bloodstream infection has been difficult to assess due to a number of factors, including confounding by underlying diseases [7,8]. We performed a prospective cohort study to gain knowledge on the etiology and antimicrobial resistance patterns of pediatric bloodstream infections and to identify microbiologic and other risk factors for fatal outcome of these infections.

## Methods

### Location and patients

The study took place from August 2001 to August 2002 at Muhimbili National Hospital, Dar es Salaam, Tanzania. A total of 1787 children (aged 0–7 years) were consecutively enrolled in a prospective cohort study of 1828 admissions. The inclusion criterion was clinical presentation suspect of systemic infection based on the presence of fever (> = 38'C), hypothermia (< 36'C) and other signs and symptoms as detailed in the WHO's IMCI Integrated Management of Childhood Illness guidelines [9] including general danger signs such as convulsions, lethargy, inability to drink or breastfeed, vomiting, and other signs of infection, such as neck stiffness, bulging fontanelles, cough, tachypnea, difficult breathing, chest in-drawings, nasal flaring, grunting, diarrhea, dehydration, ear or eye discharge, oral thrush, jaundice, enlargement of liver or spleen, lymphadenopathy, and signs of infection in the skin and umbilicus (in neonates). The attending clinician decided on inclusion of the patient and subsequently recorded clinical data using a standardized questionnaire and obtained blood for culture, malaria microscopy and HIV testing. Additionally, patients' medical records and departmental registries for admissions, discharges and deaths were reviewed.

Due to the young age of the study subjects (0 – 7 years), the parents or other accompanying, responsible family members were asked for written consent on behalf of the patient. Information was given in writing and verbally in the national language, Kiswahili. Written informed consent was obtained before taking blood for microbiological investigations, if feasible. However, in some circumstances, in the case of critically ill patients, blood specimens were taken based on verbal consent, since these investigations are strongly recommended as routine investigations in severely ill, febrile children, and since it would be inappropriate to delay management of such patients due to paperwork. The responsible family member was then approached in retrospect for written consent to use the specimen and information in the study. The responsible family member was allowed to opt out from the HIV-testing and only consent to participation in the blood culture part of the study. As far as possible, the treatment was guided by the test results. In the following, the term "suspected systemic infection" refers to all included patients in the study, and the term "laboratory-confirmed bloodstream infection" refers to growth of one or more clinically relevant bacterial or fungal isolates from blood-culture from a patient who also confirms to the inclusion criterion of suspected systemic infection. Viral and parasitic infections were not included in the definition of bloodstream infection.

Community-acquired infection was defined as bloodstream-infection with growth of pathogenic bacteria in a blood-culture obtained within the first 48 hours after admission. For classification purposes, the time to blood-culture was calculated as the time from admission to the time of receipt of blood culture in the laboratory, allowing for overnight delay in transport for cultures obtained in the evening or at night. A neonate who was born in hospital within the last 10 days was considered as having hospital-acquired infection. If a patient was discharged and re-admitted within ten days, the episode was considered a single admission. The Muhimbili University College of Health Research Ethics Committee approved the study protocol.

### Microbiologic methods

One and five ml blood from neonates and older children, respectively, were inoculated in BACTEC Myco/F lytic blood-culturing vials (Becton Dickinson, Franklin Lakes, NJ), which supports the growth of fungi and bacteria, including mycobacteria [10]. The blood-cultures were incubated for six weeks. Positive blood-cultures were subcultured on Columbia II agar base (Oxoid Ltd, Basingstoke, UK) with five percent human blood, chocolate agar and MacConckey agar (Difco/BD Diagnostic Systems, Sparks, MI, USA). The isolates were identified by standard methods [11], including the use of API20E, API20NE and API 20 AUX systems (bioMérieux SA, Marcy l'Etoile, France). Due to economic limitations, we did not perform anaerobic culture, and, in general, only a single blood culture was taken from each patient. Thus, the study was not designed to evaluate the clinical significance of coagulase-negative staphylococci (CoNS) and bacteria of doubtful or limited pathogenicity. Consequently, CoNS and probable contaminant such as diphteroids, *Bacillus *species and micrococci were not considered pathogens in this study. Enterococcal isolates were included in the study as pathogens. Cultures with polymicrobial growth were considered clinically relevant if known pathogens were among the constituent isolates.

Susceptibilities against antimicrobial agents were tested by the disk diffusion method according to the Clinical and Laboratory Standards Institute guidelines [12]. Testing for minimum inhibitory concentration (MIC) of antimicrobials was not routinely performed on all isolates. However, MIC determination by E-test (AB Biodisk, Solna, Sweden) was performed for more detailed characterization of the susceptibilities for antimicrobials in Gram-negative bacteria and enterococci. Gram-negative bacteria were investigated for extended-spectrum beta-lactamases (ESBL) with E-test, PCR and DNA sequencing as described previously [13]. Enterococcal isolates were investigated by PCR to affirm identity (*E. faecalis*, *E. faecium*) and to detect vancomycin resistance (*van*A, *van*B). We verified the identity of isolates of *S. aureus *and detected resistance to methicillin/oxacillin with a multiplex PCR targeting the *nuc *gene and the *mec*A gene [14].

Malaria testing was performed by microscopy of Giemsa-stained thick and thin drop blood smears. HIV testing was performed anonymously using a rapid test for HIV1/HIV2 antibodies (ACON HIV 1/2, ACON laboratories, Inc. San Diego, California, USA). HIV antibody-positive sera from children younger than 18 months were analyzed for HIV-1 RNA by reverse transcriptase polymerase chain reaction (RT-PCR) using primers targeting the *pol *gene, JA17 through JA20 [15], and the *vif *region OG 462/502 [16].

### Data analysis

Data were entered in a database based on the free-of-charge WHONET software for surveillance of antimicrobial resistance available as a download from the World Health Organization [17,18], and further data management was done in Filemaker Database software. Statistical analysis was performed in Stata 8 (Stata Corporation, College Station, Texas, US). Univariate assessment of risk factors for intra-hospital death was done by Fisher's exact test with a two-sided *P*-value and odds ratios and 95% confidence intervals were obtained by the 'logistic' function in Stata. Significant factors to the level of *P *< 0.2 from the univariate analysis and *a priori *important factors such as sex, age and underlying diseases were included in the multivariate analysis. Multivariate analysis was performed by automated and manual backwards step-wise logistic regression where factors with *P *> 0.2 were removed from the model. We present four logistic regression models using different subsets of the study population analyzing cases of laboratory-confirmed bloodstream infection (n = 216) as well as cases of clinically suspected systemic infection (n = 1527), and for each category we re-analyzed the data for those who had known HIV status (n = 128 and n = 790, respectively). Comparisons of medians of time variables were done by Wilcoxon rank-sum (Mann-Whitney) test.

## Results

### Patients

A total of 1787 patients with a median age of 8.5 months were admitted 1828 times. Forty-four percent (795/1787) were female. Neonates (median age 3 days, range 0–30) accounted for almost a third of the admissions (29.3%, 535/1828), and stayed a median of 6 days (range 1–53) in the hospital. Older children (median age 1 year, range 1 month – 7 years) stayed a median of 7 days (range 1–78) in the hospital.

The clinical outcome was known for 89.3% (1632/1828) of the admissions. Among the 17.0% (277/1632) who died in hospital, 22.4% (n = 62) had laboratory-confirmed bloodstream infection, 17.7% (n = 49) had malaria, 6.5% (n = 18) had concomitant laboratory-confirmed bloodstream infection and malaria-parasitemia and 52.0% (n = 144) had neither. Readmissions comprised 3.2% (n = 59) of all admissions (39 and 1 patient were readmitted once and twice, respectively, 18 patients had been admitted prior to the study).

### Antimicrobial therapy

Information on antimicrobial use was available for 85.2% (1557/1828) of the admissions. The majority of patients (93.8%) received antimicrobial therapy (Table 1) and at least two-thirds (67.2%, 1046/1557) of the patients did so before blood-culture was taken. There was no formal empirical regimen for the treatment of sepsis at the hospital, partly because of the scarcity of local studies on antimicrobial resistance of relevant bacterial isolates. However, most neonates received a regimen of ampicillin + cloxacillin + gentamicin. One-fifth of the neonates received ceftriaxone, in most cases as a second-line regimen in case of unsatisfactory response to the first regimen. In older children there was a more diverse pattern of regimens used, in most cases including one or more penicillins (ampicillin, penicillin G or cloxacillin) and either chloramphenicol or gentamicin. To some extent, accompanying localizing signs did impact on the choice of antimicrobial regimen, most notably in the case of suspected meningitis in children older than 1 month when a regimen based on chloramphenicol and a penicillin would often be used, and in the case of bloody diarrhea when erythromycin was often added. Severe malaria was treated with quinine, while less severe cases were treated with chloroquine in neonates and pyrimethamine-sulfadoxin in older children. Oral thrush was treated with nystatin mixture, but systemic antifungal drugs were rarely used. Ciprofloxacin was not used in children at the hospital.

**Table 1:** Use of antimicrobial agents in children with suspected systemic infection

	**Number of children (%) receiving drug**			
**Age of child on blood culture**	**0–6 days**	**7–30 days**	**1 month – 7 years**	**Total**

**No of patients**	**n = 261**	**n = 190**	**n = 1106**	**n = 1557**

*Any antibacterial*	*257 (98.5)*	*186 (97.9)*	*1017 (92.0)*	*1460 (93.8)*
- Penicillin	-	-	301 (27.2)	301 (19.3)
- Ampicillin/amoxicillin	244 (93.5)	165 (86.8)	589 (53.3)	998 (64.1)
- Cloxacillin	244 (93.5)	165 (86.8)	354 (32.0)	763 (49.0)
- Cephalexin	-	-	27 (2.4)	27 (1.7)
- Cefuroxime	-	-	37 (3.3)	37 (2.4)
- Ceftriaxone	34 (13.0)	51 (26.8)	118 (10.7)	203 (13.0)
- Chloramphenicol	-	2 (1.1)	416 (37.6)	418 (26.8)
- Gentamicin	250 (95.8)	173 (91.1)	504 (45.6)	927 (59.5)
- Amikacin	-	1 (0.5)	25 (2.3)	26 (1.7)
- Co-trimoxazole	-	3 (1.6)	129 (11.7)	132 (8.5)
- Erythromycin	-	2 (1.1)	44 (4.0)	46 (3.0)
- Azithromycin	-	-	23 (2.1)	23 (1.5)
- Nalidixic acid	-	-	4 (0.4)	4 (0.3)
- Metronidazole	-	-	22 (2.0)	22 (1.4)
*TB medicines*	-	-	71(6.4)	71(4.6)
*Any antimalarial*	19(7.3)	*23 (12.1)*	*784 (70.9)*	*826 (53.1)*
- Quinine	6 (2.3)	16 (8.4)	636 (57.5)	658 (42.3)
- Sulfadoxine-pyrimethamine	-	1 (0.5)	192 (17.4)	193 (12.4)
- Chloroquine	15 (5.7)	9 (4.7)	7 (0.6)	31 (2.0)
- Amodiaquine	-	-	12 (1.1)	12 (0.8)
- Artesunate	-	-	1 (0.1)	1 (0.1)
*Mebendazole*	-	-	44(4.0)	44(2.8)
*Nystatin (oral)*	8(3.1)	13(6.8)	*195 (17.6)*	*216 (13.9)*
*Systemic antifungal*	-	-	8(0.7)	8(0.5)

### Types of bloodstream infections

The incidence of laboratory-confirmed bloodstream infection was 13.9% (255/1828) of all admissions, 15.9% (85/535) among neonates and 13.1% (170/1293) among older children. A single pathogen was recovered from 224 children (12.3%), while 31 (1.7%) had polymicrobial infection with two (n = 26), three (n = 2) or four isolates (n = 3). In total, 294 pathogenic bacterial and fungal isolates were recovered (Table 2). Among all laboratory-confirmed bloodstream infections, half (128/255) were defined as potentially hospital-acquired. Salmonella and *Escherichia coli *were the most common isolates in community-acquired infections, and klebsiella and *Staphylococcus aureus *were the most common in hospital-acquired infections (Table 2). Klebsiella was, by far, the most common cause of neonatal bloodstream infection, particularly in early-onset infection (0–6 days of age) where it responsible for approximately one-third of the cases (Table 3). In children older than 1 month of age, salmonellae were the most frequently isolated pathogens (Table 3).

**Table 2:** Frequency (percentage) of bacterial and fungal pathogens* cultured from community-acquired† and hospital-acquired‡ bloodstream infection

**Organism**	**CA† **	**HA‡ **	**Total**
*Klebsiella *spp.	19 (12.3)	34 (24.5)	53 (18.0)
- *Klebsiella pneumoniae*	17	31	48
Salmonellae	27 (17.4)	12 (8.6)	39 (13.3)
- *Salmonella *serovar Typhii	1	1	2
- *Salmonella *serovar Enteritidis	14	6	20
- *Salmonella *serovar Typhimurium	11	5	16
- *Salmonella *serovar Newport	1	0	1
*Escherichia coli*	24 (15.5)	13 (9.4)	37 (12.6)
*Enterobacter *spp.	5 (3.2)	4 (2.9)	9 (3.1)
- *Enterobacter cloacae*	2	4	6
- Other *Enterobacter *spp.	3	0	3
Other *Enterobacteriaceae*	4 (2.6)	2 (1.4)	6 (2.0)
*- Pantoea *spp.	2	0	2
*- Serratia marcescens*	0	1	1
*- Shigella flexnerii*	1	0	1
*- Citrobacter freundii*	1	0	1
*- Proteus mirabilis*	0	1	1
**Total *Enterobacteriaceae***	**79 (51.0)**	**65 (46.8)**	**144 (49.0)**
*Acinetobacter *spp.	3 (1.9)	9 (6.5)	12 (4.1)
- *Acinetobacter baumannii*	0	5	5
- *Acinetobacter lwoffii*	3	2	5
- *Acinetobacter *spp.	0	2	2
*Pseudomonas aeruginosa*	7 (4.5)	6 (4.3)	13 (4.4)
Other Non-*Enterobacteriaceae*	6 (3.9)	5 (3.6)	11 (3.7)
*Pseudomonas *spp.	4	1	5
*Sphingomonas paucimobilis*	2	0	2
*Chryseobacterium *spp.	0	2	2
*Moraxella *spp.	0	2	2
**Total non-*Enterobacteriaceae*GNR**	**16 (10.3)**	**20 (14.4)**	**36 (12.2)**
GNR not further specified	5 (3.2)	2 (1.4)	7 (2.4)
**Total GNR**	**100 (64.5)**	**87 (62.6)**	**187 (63.6)**
*Staphylococcus aureus*	13 (8.4)	17 (12.2)	30 (10.2)
Enterococci	24 (15.5)	19 (13.7)	43 (14.6)
- *Enterococcus faecium*	12	9	21
- *Enterococcus faecalis*	9	6	15
- *Enterococcus *spp.	3	4	7
Streptococci	3 (1.9)	5 (3.6)	8 (2.7)
- Group B streptococci	1	3	4
*- Streptococcus viridans*	1	2	3
- Streptococci, not further identified	1	0	1
**Total Gram-positive**	**40 (25.8)**	**41 (29.5)**	**81 (27.6)**
*Mycobacterium tuberculosis*	0 (0.0)	1 (0.5)	1 (0.3)
*Candida *spp.	15 (9.7)	10 (7.2)	25 (8.5)
**Total**	**155 (100.0)**	**139 (100.0)**	**294 (100.0)**

GNR, Gram-negative rods. *The percentage refers to the proportion of all pathogenic bacterial and fungal isolates. Anaerobes, coagulase-negative staphylococci and bacterial isolates of uncertain pathogenicity were not included. † CA: Community-acquired infection, i.e. blood-culture obtained ≤ 48 hours from of admission. ‡ Hospital-acquired infection: Blood-culture obtained > 48 hours from admission.

**Table 3:** Episodes of laboratory-confirmed bloodstream infection in different age groups of children

Organism*	**Early onset† N (%)**	**Late onset‡ N (%)**	**Older§ N (%)**	**Total N (%)**
*Klebsiella *spp.	17 (31.5)	7 (22.6)	12 (7.1)	36 (14.1)
Salmonellae	0 (0.0)	1 (3.2)	32 (18.8)	33 (12.9)
*Escherichia coli*	6 (11.1)	3 (9.7)	19 (11.2)	28 (11.0)
*Enterobacter *spp.	4 (7.4)	1 (3.2)	2 (1.2)	7 (2.7)
Other *Enterobacteriaceae*	1 (1.9)	0 (0.0)	1 (0.6)	2 (0.8)
**Total *Enterobacteriaceae***	**28 (51.9)**	**12 (38.7)**	**66 (38.8)**	**106 (41.6)**
*Acinetobacter *spp.	1 (1.9)	0 (0.0)	10 (5.9)	11 (4.3)
*Pseudomonas aeruginosa*	0 (0.0)	0 (0.0)	9 (5.3)	9 (3.5)
Other non-*Enterobacteriaceae*	4 (7.4)	0 (0.0)	5 (2.9)	9 (3.5)
**Total non-*Enterobacteriaceae***	**5 (9.3)**	**0 (0.0)**	**24 (14.1)**	**29 (11.4)**
GNR not further specified	1 (1.9)	1 (3.2)	5 (2.9)	7 (2.7)
**Total GNR**	**34 (63.0)**	**13 (41.9)**	**95 (55.9)**	**142 (55.7)**
*Staphylococcus aureus*	6 (11.1)	5 (16.1)	15 (8.8)	26 (10.2)
Enterococci	3 (5.6)	4 (12.9)	23 (13.5)	30 (11.8)
Group B streptococci	2 (3.7)	1 (3.2)	0 (0.0)	3 (1.2)
Other *Streptococcus*	1 (1.9)	0 (0.0)	2 (1.2)	3 (1.2)
**Total Gram positive**	**12 (22.2)**	**10 (32.3)**	**40 (23.5)**	**62 (24.3)**
*Mycobacterium tuberculosis*	0 (0.0)	0 (0.0)	1 (0.6)	1 (0.4)
*Candida *spp.	1 (1.9)	3 (9.7)	15 (8.8)	19 (7.5)
**Polymicrobial infections**	**7 (13.0)**	**5 (16.1)**	**19 (11.2)**	**31 (12.2)**
**Total**	**54 (100.0)**	**31 (100.0)**	**170 (100.0)**	**255 (100.0)**

GNR, Gram-negative rods. *The percentage refers to the proportion of all episodes of laboratory-confirmed bloodstream infection. Anaerobes, coagulase-negative staphylococci and bacterial isolates of uncertain pathogenicity were not included. † Early-onset = age 0–6 days. ‡ Late-onset = age 7–30 days. † Older = 1 month – 7 years.

**Figure 1:**
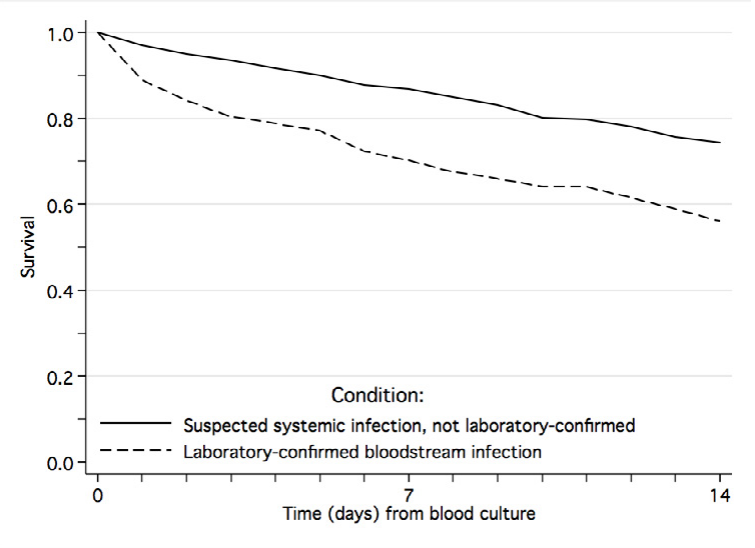
Survival from laboratory-confirmed bloodstream infection and clinically suspected systemic infection.

**Figure 2:**
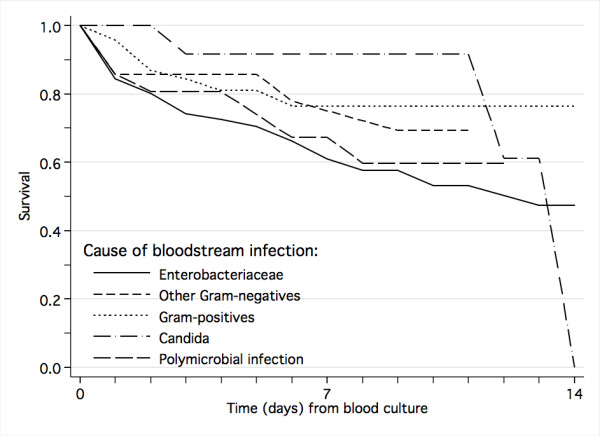
Impact of causative organism on survival from laboratory-confirmed bloodstream infection.

Among all children with suspected systemic infection, those with laboratory-confirmed bloodstream infection had three times increased risk of dying (Table 4, Figure 1), and among those who survived, the duration of hospital stay was significantly longer than for children without laboratory-verified bloodstream infection (median 8 versus 6 days, *P *< 0.001). Univariate and multivariate analysis of risk factors for death are shown in Table 4 and 5, respectively. The mortality rate from Gram-negative bloodstream infection (45.6%) was more than twice that of malaria (20.2%) and Gram-positive bloodstream infection (16.7%). Positive blood-culture with *Enterobacteriaceae*, other Gram-negative bacteria and candida were independent risk factors for fatal outcome (Table 5, Figure 2). In a separate logistic regression model examining only the pathogen involved, the significant risk factors for death were, in descending order of odds ratio, growth of *Enterobacter *spp. (odds ratio 10.5, *P *= 0.007), *Pseudomonas aeruginosa *(9.7, *P *< 0.001), *Salmonella *Typhimurium (6.4, *P *= 0.001), *E. coli *(5.0, *P *< 0.001), klebsiella (4.5, *P *< 0.001), and candida (2.5, *P *= 0.049) (data not shown in table). Growth of *Salmonella *Enteritidis, *Acinetobacter *spp., *E. faecalis*, *E. faecium *and *S. aureus *were not significant risk factors for death.

**Table 4:** Univariate analysis of risk factors for intrahospital death among 1632 children* with suspected systemic infection

**Risk factor**	**CFR % (n)**	**OR**	**95% CI**	***P***
Overall	17.0 (277/1632)			
Male sex	15.5 (143/920)	0.79	0.61 to 1.03	0.084
Neonate (< 1 m)	14.8 (70/473)	0.80	0.59 to 1.07	0.146
				
**Underlying conditions**				
Malnutrition	29.3 (71/242)	2.37	1.73 to 3.25	< 0.001
HIV infection (laboratory confirmed)	31.7 (46/145)	2.40	1.60 to 3.59	< 0.001
*Other infectious underlying conditions*	30.6 (33/108)	2.29	1.48 to 3.52	< 0.001
- Tuberculosis	30.5 (29/95)	2.26	1.43 to 3.58	0.001
- Hepatitis	44.4 (4/9)	3.92	1.04 to 14.67	0.052
- Congenital syphilis	50.0 (1/2)	4.85	0.30 to 77.79	0.313
*Other non-infectious underlying conditions*	17.1 (20/117)	1.00	0.60 to 1.64	1.000
- Sickle cell disease	3.0 (1/33)	0.15	0.02 to 1.09	0.032
- Neoplasia	60.0 (3/5)	7.32	1.22 to 44.05	0.038
- Congenital heart disease	27.3 (9/33)	1.77	0.82 to 3.83	0.156
				
Malaria (laboratory confirmed)	20.2 (67/331)	1.29	0.95 to 1.76	0.102
				
*Growth in blood culture of:*				
Any pathogen	34.9 (80/229)	3.29	2.41 to 4.48	< 0.001
Any GNR	43.5 (64/147)	4.60	3.22 to 6.60	< 0.001
Any *Enterobacteriaceae*	45.6 (52/114)	4.82	3.25 to 7.15	< 0.001
- *E. coli*	50.0 (15/30)	5.11	2.47 to 10.59	< 0.001
- *Klebsiella *spp.	48.9 (22/45)	5.00	2.74 to 9.10	< 0.001
- All non-typhoid Salmonellae	36.4 (12/33)	2.88	1.40 to 5.92	0.007
- *Salmonella *Enteritidis	21.1 (4/19)	1.31	0.43 to 3.97	0.548
- *Salmonella *Typhimurium	53.9 (7/13)	5.83	1.94 to 17.48	0.003
- *Enterobacter *spp.	71.4 (5/7)	12.43	2.40 to 64.43	0.002
Any non-*Enterobacteriaceae *GNR	40.6 (13/32)	3.46	1.69 to 7.10	0.001
- *Pseudomonas aeruginosa*	66.7 (8/12)	10.04	3.00 to 33.59	< 0.001
- *Acinetobacter *spp.	9.1 (1/11)	0.49	0.06 to 3.82	0.702
Gram-positive pathogen†	16.7 (11/66)	0.98	0.50 to 1.89	1.000
- Enterococci	19.4 (7/36)	1.19	0.51 to 2.73	0.655
- *E. faecium*	6.7 (1/15)	0.35	0.05 to 2.65	0.490
- *E. faecalis*	28.6 (4/14)	1.97	0.61 to 6.33	0.275
- *S. aureus*	7.1 (2/28)	0.37	0.09 to 1.58	0.208
Candida	33.3 (8/24)	2.49	1.05 to 5.87	0.049
Polymicrobial infection	41.4 (12/29)	3.56	1.68 to 7.55	0.002
				
Inappropriate antimicrobial therapy due to:				
- ESBL-producing, multiresistant isolates	71.4 (15/21)	12.87	4.95 to 33.48	< 0.001
- Other bacterial resistance (non-ESBL)	37.8 (17/45)	3.14	1.69 to 5.83	0.001
- Any bacterial resistance (ESBL + other)	47.7 (31/65)	5.01	3.02 to 8.31	< 0.001
- Any antimicrobial resistance (including fungi)	43.8 (39/89)	4.39	2.82 to 6.83	< 0.001
				
Hospital-acquired infection	35.5 (44/124)	3.01	2.03 to 4.46	< 0.001
Readmission stay	15.2 (14/92)	0.87	0.49 to 1.56	0.775

CFR, case-fatality rate; OR, odds ratio; 95%CI: 95% confidence interval; *P*, p-value calculated with two-sided Fisher's exact test; GNR, Gram-negative rod; ESBL, extended-spectrum beta-lactamase. *The outcome was known for 1632 among a total of 1828 admissions. † Anaerobes, coagulase-negative staphylococci and bacterial isolates of uncertain pathogenicity were not included. ‡ Blood culture obtained > 2 days after admission.

**Table 5:** Logistic regression analysis of risk factors for intra-hospital death from laboratory-confirmed bloodstream infection and clinically suspected systemic infection

**Characteristic**	**Laboratory-confirmed bloodstream infection**		**Clinically suspected systemic infection**	
**HIV-status analyzed**	**No**	**Yes**	**No**	**Yes**

**No. of observations**	**n = 216**	**n = 128**	**n = 1527**	**n = 790**

	OR (95%CI)	OR (95%CI)	OR (95%CI)	OR (95%CI)
Male sex	*	*	0.8 (0.60–1.05)	*
Neonate (≤ 1 month)	*	*	*	0.7 (0.45–1.15)
Malnutrition	1.9 (0.95–3.88)	*	2.1 (1.47–2.90)‡	1.6 (0.96–2.55)
HIV infection	NA	3.4 (1.22–9.40)†	NA	2.1 (1.29–3.26)†
Other underlying infectious disease	*	*	1.8 (1.13–2.87)†	*
Underlying non-infectious disease	*	*	*	*
Malaria	*	*	*	*
Hospital-acquired infection	*	*	*	*
Polymicrobial infection	*	*	*	*
Growth in blood-culture of:				
- *Enterobacteriaceae*	3.5 (1.71–7.03)†	5.6 (2.06–14.95)†	3.3 (2.09–5.34)‡	4.2 (2.27–7.65)‡
- Non *-Enterobacteriaceae*	2.4 (0.92–6.13)	4.0 (0.99–16.45)	2.4 (1.02–5.46)†	2.7 (0.80–8.81)
- *Candida *spp.	2.6 (0.91–7.29)	2.9 (0.70–12.09)	2.5 (1.02–6.03)†	*
Inappropriate antimicrobial treatment	2.1 (1.09–4.16)†	2.3 (0.95–5.58)	2.1 (1.14–3.93)†	1.7 (0.76–3.73)

OR, odds ratio; 95%CI, 95% confidence interval. *Variables with *P *> 0.2 were removed from the models. Statistically significant risk factors are marked † *P *< 0.05 and ‡ *P *< 0.001.

### Malaria

Malaria slides were positive in 21.6% (354/1638). Malaria parasitemia was neither associated with HIV-infection (*P *= 0.227), nor bloodstream infection caused by any pathogen (*P *= 0.730), Gram-negative bacteria (*P *= 0.296), *Enterobacteriaceae *(*P *= 0.351), *E. coli *(*P *= 0.516), klebsiella (*P *= 1.000), non-typhoid salmonella (*P *= 0.664). The case-fatality rate was not significantly higher in patients with malaria parasitemia (20.2%) compared to those without (16.4%, *P *= 0.102). However, in patients with positive malaria slides, mortality was almost three times higher among those with concomitant Gram-negative bloodstream infection (45.7% versus 17.2%, *P *< 0.001).

### Susceptibility to antimicrobial agents

*Enterobacteriaceae *displayed high rates of resistance to commonly used antimicrobials (Table 6). Only 20% of community-acquired *Enterobacteriaceae *isolates was sensitive to ampicillin, while two-thirds were sensitive to gentamicin. Two-thirds of community-acquired salmonellae were sensitive to chloramphenicol, while half are resistant to ampicillin and to co-trimoxazole. Isolates of *Enterobacteriaceae *were almost uniformly sensitive to ciprofloxacin. *P. aeruginosa *isolates were commonly sensitive to anti-pseudomonas drugs such as ceftazidime, ciprofloxacin and tobramicin (Table 7). ESBL phenotype was found in 18% of the *Enterobacteriaceae *isolates (*E. coli *9/37, klebsiella 9/53, *Enterobacter *spp. 5/9, salmonella 1/39 and *Pantoea *spp 2/2) involving TEM-63, SHV-2a, SHV-12 and CTX-M-15 genotypes [13], and in 3 isolates of non-*Enterobacteriaceae *(one *Acinetobacter *spp. and the 2 *Chryseobacterium *spp.). ESBL-producing isolates were resistant to almost all tested antimicrobials except for ciprofloxacin and meropenem.

**Table 6:** Antimicrobial susceptibility (percentage) of *Enterobacteriaceae *isolates causing bloodstream infection

**Antimicrobial**	**Klebsiella**		**Salmonella**		***E. coli***		Other*		**Total**	
	CA 19	HA 34	CA 27	HA 12	CA 24	HA 13	CA 9	HA 6	CA 79	HA 65

Ampicillin	0	0	52	33	4	15	11	33	20	12
Amoxicillin-clavulanate	53	62	70	67	75	31†	11	17	61	52
Cefuroxime	74	85	93	100	88	54†	56	50	82	78
Ceftazidime	79	85	96	100	88	46†	56	50	85	77
Cefotaxime	78	85	96	100	88	50†	56	67	84	80
Meropenem	100	100	100	100	100	100	100	100	100	100
Gentamicin	53	53	74	67	71	54	33	50	63	55
Doxycycline	58	71	85	75	13	23	33	17	51	57
Co-trimoxazole	37	6†	52	33	13	23	22	50	33	18
Chloramphenicol	47	56	85	67	33	46	22	50	53	55
Ciprofloxacin	100	100	100	100	92	92	89	100	96	98

CA, community-acquired infection; HA, hospital-acquired infection. *Other isolates include *Enterobacter *spp., (9), *Pantoea *spp. (2), *Serratia marcescens *(1), *Shigella flexneri *(1), *Citrobacter freundii *(1), *Proteus mirabilis *(1). † Statistically significant difference in susceptibility among hospital-acquired and community-acquired isolates, p < 0.05 by two-sided Fisher's exact test.

**Table 7:** Antimicrobial susceptibility (percentage) of non-*Enterobacteriaceae *Gram-negative isolates causing bloodstream infection

**Antimicrobial**	***P. aeruginosa***		***Acinetobacter*spp.**		Other*		**Total**	
	CA 7	HA 6	CA 3	HA 9	CA 6	HA 5	CA 16	HA 20

Ampicillin	0	0	0	0	17	0	7	0
Amoxicillin-clavulanate	17	20	67	33	67	0	47	22
Cefuroxime	20	0	67	22	67	25	50	17
Ceftazidime	100	100	67	78	83	50	88	79
Meropenem	100	100	100	100	50	50	85	88
Gentamicin	86	83	33	56	50	50	63	63
Tobramicin	86	100	-	-	-	-	-	-
Doxycycline	0	0	67	56	100	50	54	47
Co-trimoxazole	0	0	0	0	33	50	14	13
Chloramphenicol	20	0	33	22	67	0	43	13
Ciprofloxacin	100	100	100	78	100	100	100	89

CA, community-acquired infection; HA, hospital-acquired infection; "-", not done. *Other isolates include other *Pseudomonas *spp., (5), *Sphingomonas paucimobilis *(2), *Chryseobacterium *spp. (2), *Moraxella *spp. (2).

The majority of *S. aureus *isolates were sensitive to commonly used anti-staphylococcal agents, including cloxacillin and gentamicin (Table 8). Among the three isolates phenotypically resistant to oxacillin, only one was available for confirmatory detection of the *mec*A gene by PCR. The child with confirmed MRSA genotype died, the two other survived. Ten of 21 *E. faecium *isolates showed combined resistance to ampicillin and gentamicin (high-level), but only one patient with bloodstream infection caused by these organisms died. This combined resistance trait occurred in both community-acquired (4/12) and hospital-acquired infections (6/9), and the difference was not statistically different (*P *= 0.198). Six of 15 *E. faecalis *isolates were high-level gentamicin-resistant (but not ampicillin-resistant) and were involved in the death of two patients, among whom one had community-acquired infection. The majority of candida isolates were susceptible to fluconazole (96%) and amphotericin B (87%).

**Table 8:** Antimicrobial susceptibility (percentage) of Gram-positive bacteria causing bloodstream infection

**Drug**	***S. aureus***		E. faecium*		E. faecalis*	
	CA 13	HA 17	CA 12	HA 9	CA 9	HA 6

Penicillin	0	0	10	0	17	75
Ampicillin*	-	-	25	11	100	100
Amoxicillin-clavulanate	77	50	70	11†	100	100
Cloxacillin	92	88	-	-	-	-
Cefuroxime	92	88	-	-	-	-
Ceftazidime	85	80	-	-	-	-
Meropenem	100	100	-	-	-	-
Gentamicin*	100	81	67	33	56	67
Vancomycin*	100	100	100	100	100	100
Erythromycin	100	71	0	0	22	33
Doxycycline	62	35	9	0	25	0
Co-trimoxazole	69	63	27	0	67	40
Chloramphenicol	85	73	50	11	38	33
Ciprofloxacin*	77	81	42	22	100	100
Linezolid*	-	-	100	100	100	100
Quinupristin-dalfopristin*	-	-	100	100	0	17

CA, community-acquired infection; HA, hospital-acquired infection; "-", not done. *E-test was used for susceptibility testing of enterococci against ampicillin, ciprofloxacillin, linezolid, quinupristin-dalfopristin, vancomycin and high-level gentamicin resistance. † Statistically significant difference in susceptibility among hospital-acquired and community-acquired isolates, p < 0.05 by two-sided Fisher's exact test.

Antimicrobial treatment prior to blood culture was significantly associated with resistance to co-trimoxazole (83% versus 69%, *P *= 0.036) and chloramphenicol (59% versus 42%, *P *= 0.035) in Gram-negative isolates as a group, with resistance to erythromycin (36% versus 0%, *P *= 0.014) and chloramphenicol (46% versus 0%, *P *= 0.005) in *S. aureus *and chloramphenicol in *E. faecalis *(89% versus 20%, *P *= 0.023). In addition, all three MRSA isolates were from patients who had received previous antimicrobial treatment, but this was not statistically significant.

Hospital-acquisition of infection was significantly associated with resistance to amoxicillin-clavulanate and cephalosporins in *E. coli *and with co-trimoxazole-resistance in klebsiella. The percentage of bloodstream infection episodes caused by an ESBL-producing organism was not significantly different among community-acquired (7%, 9/127) and hospital-acquired infections 13% (16/128, *P *= 0.206). Patients with hospital-acquired laboratory-verified bloodstream infection did not receive inappropriate antimicrobial treatment more often (41%) than those with community-acquired infection (42%, *P*≅0.892).

**Figure 3:**
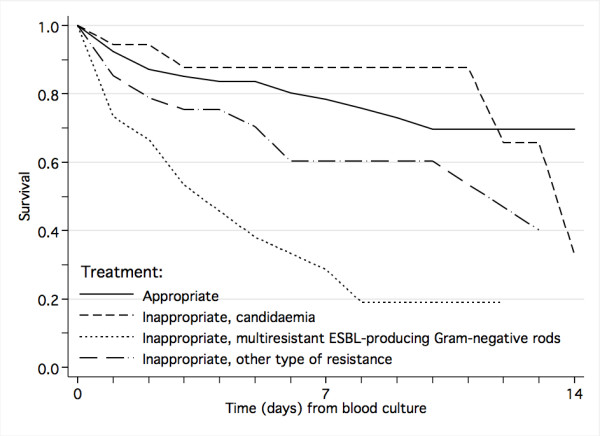
Impact of antimicrobial resistance on survival from laboratory-confirmed bloodstream infection.

Inappropriate antimicrobial treatment was a risk factors for death irrespectively of whether controls were patients with clinically suspected infection or laboratory-confirmed bloodstream infection (Table 5, Figure 3). In those who survived, inappropriate antimicrobial treatment for the bloodstream-infection was associated with increased duration of hospital stay (median 8 days versus 6 days, *P *= 0.043).

**Figure 4:**
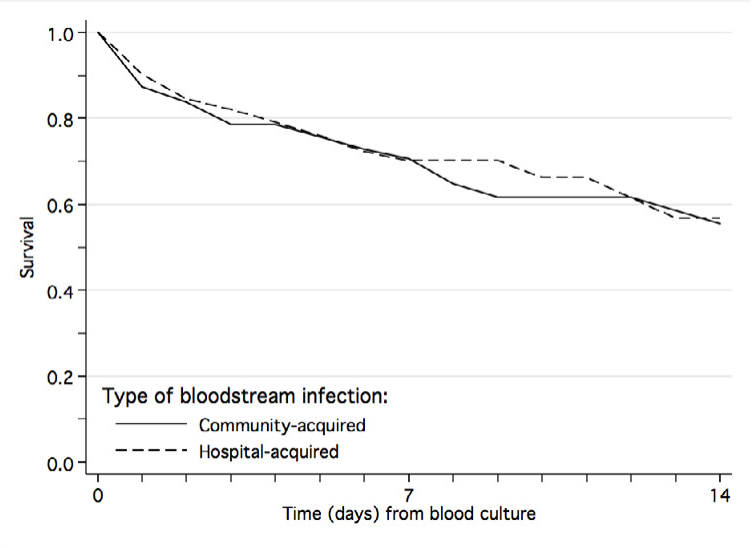
Survival from community-acquired and nosocomial laboratory-confirmed bloodstream infection.

Hospital-acquired infection was associated with increased duration of hospitalization (median 10 days versus 6 days, *P *< 0.001), but was not a significant risk factor for fatal outcome in the multivariate analysis (Table 5, Figure 4).

### HIV-infection

Fifty-one percent (911/1787) of the study subjects were examined for HIV antibodies and 24.0% (219/911) were positive. The HIV-1 PCR was positive in 55.4% (82/148) of children younger than 18 months and 8.9% (5/56) of neonates. Thus, the HIV-1 prevalence was 1.7% (5/302) for neonates, 24.4% (147/603) for older children, and the combined overall prevalence was 16.8% (152/905).

**Figure 5:**
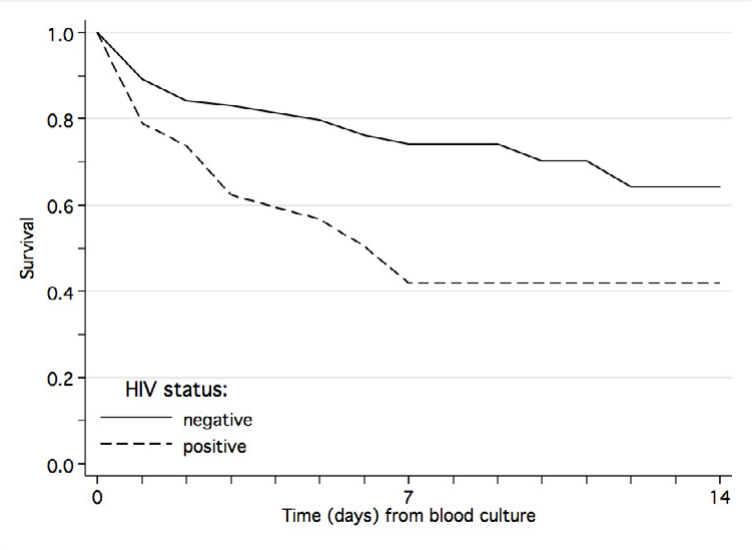
Impact of HIV co-infection on survival from laboratory-confirmed bloodstream infection.

Laboratory-confirmed bloodstream infection was not significantly more frequent in HIV-positive children (17.5, 28/160) than in HIV-negative ones (15.5%, 119/766, *P *= 0.552). However, bloodstream infection was more frequently caused by non-typhoid salmonella in HIV-positive children (25%, 7/28) than in HIV-negative children (8.4%, 10/119, *P *= 0.022). There was no such association between HIV-infection and bloodstream infection caused by *E. coli *(17.9% versus 10.9%, *P *= 0.339), ESBL-producing organisms (17.9% versus 10.1%, *P *= 0.321), candida (10.7% versus 10.9%, *P *= 1.000) or other pathogens, except for bloodstream infection caused by klebsiella, which was negatively associated with HIV infection (7.1% versus 33.6%, *P *= 0.005). HIV-positive children as a group did not receive more antimicrobial therapy (97.2% versus 93.4%, *P *= 0.082). However, the given antimicrobial therapy for those with bloodstream infection was inappropriate more often than in HIV-negative children (64.0% versus 40.2%, *P *= 0.044). HIV-infection was a risk factor for death (Table 5, Figure 5), and associated with significantly longer hospital stay in those who survived (median 9 versus 6 days, *P *< 0.001).

### Malnutrition and other underlying conditions

**Figure 6:**
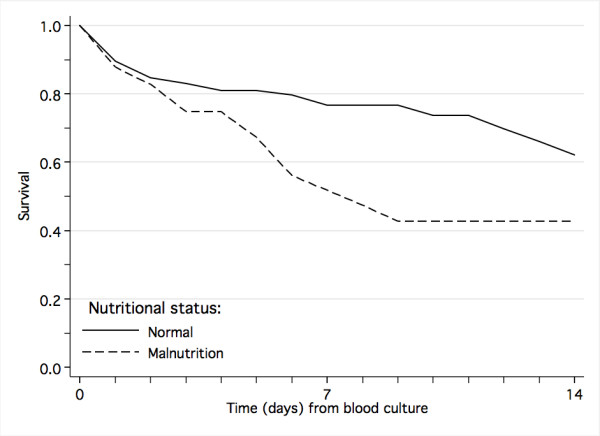
Impact of nutritional status on survival from laboratory-confirmed bloodstream infection.

One sixth (15.5%, 248/1603) of the patients were malnourished. Malnutrition was a risk factor for death (Table 5, Figure 6) and, in those who survived, it was associated with prolonged hospital stay (median 10 versus 6 days, *P *< 0.001). Other underlying diseases were grouped into infectious diseases, including tuberculosis (n = 97), hepatitis (n = 9) and congenital syphilis (n = 2) and non-infectious conditions, including sickle cell disease (n = 36), cancer (n = 5), congenital heart disease (n = 38), asthma (n = 5), Down's syndrome (n = 11), cerebral palsy (n = 25), congenital malformations (n = 21), epilepsy (n = 10) and hemophilia (n = 2). Other underlying infectious conditions than HIV were a significant risk factor for fatal outcome among all patients with clinically suspected systemic infection.

## Discussion

Considering the frequent use of antimicrobials prior to blood-culture, the study probably underestimates the incidence of bloodstream infection, particularly episodes caused by fastidious organisms such as pneumococci and other organisms susceptible to commonly used antimicrobials. Nevertheless, the observed incidence of laboratory-confirmed bloodstream infection was high (13.9%) and comparable to that of malaria (21.6%). The therapeutic dilemma is evident, since bloodstream infection and malaria are difficult to distinguish based on clinical presentation [4,5]. While mortality was similar in bloodstream infection and malaria in a study from Rwanda in the 1980s [19], bloodstream infection carried much higher mortality than malaria in our study. As reported recently from Kenya [3], deaths from bloodstream infection outnumbered malaria deaths. The three times higher mortality among malaria-parasitemic patients with concomitant Gram-negative bloodstream infection underlines the relative importance of bloodstream infections. Prompt antimicrobial treatment is imperative for the survival of patients with bacterial bloodstream infection [20]. However, the impact of antimicrobial resistance on the clinical outcome has not been firmly ascertained, particularly in developing country settings [6-8,21,22]. The present study confirms that inappropriate treatment of bloodstream infections due to antimicrobial resistance predicts fatal outcome independently of underlying diseases, and is associated with longer duration of hospital stay in those who survive. While morbidity and cost are more sensitive measures of the impact of antimicrobial resistance in rich countries [7], our study demonstrates that the toll of antimicrobial resistance in Sub-Saharan Africa is quantifiable in loss of human lives.

The high rates of resistance in Gram-negative bacteria confirm previous findings [23]. However, the frequent use of antimicrobials prior to blood culture may have biased the findings by lowering the detection rate for organisms susceptible to commonly used drugs. It is grave that half of the klebsiella isolates, which are inherently resistant to ampicillin, are also resistant to gentamicin, since these two drugs are the most frequently used drugs for the treatment of bloodstream infections. The high incidence and case-fatality rate of klebsiella bloodstream infection in our study supports the findings by Zaidi and colleagues that klebsiella infections may be responsible for more than 0.3 million yearly neonatal deaths globally [22]. As reported elsewhere [19,24,25], salmonella was the most frequent cause of bloodstream infections in older children. Non-typhoid salmonella cause a wide range of manifestations from self-limiting gastroenteritis to systemic infections such as bloodstream infections and sometimes meningitis associated with high mortality [26,27]. Bloodstream infection caused by *Salmonella *Typhimurium was associated with fatal outcome with comparable case-fatality rates as other major *Enterobacteriaceae *species, while bloodstream infection caused by *S*. Enteritidis was associated with lower case-fatality rates in the same range as Gram-positive bloodstream infections. The relatively low case-fatality rate from bloodstream infection caused by *S. aureus *reflects the frequent use of cloxacillin and the near absence of methicillin-resistance. Bloodstream infection caused by *E. faecium *resistant to ampicillin and gentamicin is a potentially serious problem considering the widespread reliance on these drugs for treatment. The relatively low case-fatality rate from bloodstream infection caused by these organisms may reflect an inherently less virulent nature. However, it is also possible that some of the *E. faecium *isolates may have been skin contaminants, in which case the study may overestimate the incidence of bloodstream infection caused by this organism.

Hospital-acquired infection was associated with increased rate of resistance to cephalosporins in *E. coli *and co-trimoxazole in klebsiella, but it was not a risk factor for fatal outcome. Possibly, the definition used for hospital-acquired infection may have been too strict in the current study setting. The classification as hospital-acquired depended partly on the difference in time from admission to time of receiving the blood culture in the laboratory. Thus, any delay in obtaining or transporting blood-cultures may have led to some cases of community-acquired infection being wrongly classified as hospital-acquired. On the other side, it is less likely that cases of community-acquired infection have been misclassified as such, although we cannot rule out the possibility of underreporting of previous hospitalization.

Candida bloodstream infection was more frequent than reported from comparable low-income countries [22] and more in line with the trend in developed countries [28]. Candida bloodstream infection was not associated with HIV-infection or malnutrition. Candida was as common as a cause of community-acquired bloodstream infections (10.2%) as hospital-acquired ones (9.4%, *P *= 0.837). The rare use of systemic antifungals may partly explain the high case-fatality rate associated with candida bloodstream infection.

The HIV prevalence (16.8%) was higher than the country average (7%) [29] and the estimate for Dar es Salaam (10.8%) [30], but lower than in a study of children admitted to the same hospital in 1995–96 (19.2%) [31], in which HIV status was verified by p24 antigen detection. The selection of study population (hospitalized children with fever) and the refusal by some parents to test their child may have influenced the HIV prevalence estimate.

Co-trimoxazole prophylaxis against *Pneumocystis jirovecii *pneumonia has been linked to resistance to this drug in HIV infected individuals [32-34]. In our study, HIV-positive subjects did receive inappropriate antibacterial therapy more frequently than HIV-negative, however, there was no significantly difference in resistance traits when analyzing for specific organism groups. Malnutrition predisposes patients for bacterial infection and is an underlying factor in more than half of under-five deaths [2]. Our study support the findings of previous studies that both malnutrition and HIV infection affect adversely the outcome of bloodstream infection [2,34,35]. The association between salmonella infection in HIV-infection is well-known and has been attributed to a deficiency in the immune system that permits intracellular survival of the bacteria despite presumed adequate treatment [24,25,36]. While other infections such as *Pneumocystis jirovecii *pneumonia are important causes of death in HIV-infected children [37], our study suggest that bloodstream infection may contribute to the excess mortality in HIV-infected children in Sub-Saharan Africa [38].

In contrast to studies on adults from Tanzania and elsewhere [39], we isolated *Mycobacterium tuberculosis *from blood-culture from only one of 97 children with clinical tuberculosis. This finding indicates that blood-culture is a poor diagnostic tool for pediatric tuberculosis and underlines the experience from other studies that it is difficult to establish microbiological evidence to support the diagnosis of tuberculosis in children [40].

One limitation of the study is that clinical and outcome data were missing for some patients. Furthermore, we performed neither anaerobic culture nor repeat cultures to assess the significance of coagulase-negative staphylococci. The frequent use of antimicrobials before blood-culture and the small blood volume cultured, particularly from neonates (1 ml), likely precluded the detection of pathogens, particularly fastidious organisms. The blood-culture system supported growth of pneumococci during quality testing in our laboratory. While available documentation by the start of our study supported use of the blood-culture system [10,41], a later study indicated that it was not optimal for detection of pneumococci and *S. aureus *[42]. Sub-culturing on human blood agar plates may have interfered with the detection of pneumococci in the study. Human blood agar, prepared from expired banked blood, is widely used for bacterial isolation in developing countries, but has recently been demonstrated to be inferior to animal blood agars (sheep or horse blood) in detecting growth of common bacteria such as pneumococci, *S. pyogenes *and *S. aureus*. The use of human blood agar leads to reduced bacterial colony size, altered colony morphology and poor hemolysis [43]. The reasons for this are not fully understood, but may include remnants of antimicrobials consumed by the blood donor, antibodies or other unknown factors. While citrate in banked human blood has been considered a possible explanation for the inferiority of human blood agar for bacterial culture, Russell and colleagues found citrated sheep blood agar to be a practical and superior alternative to human blood agar [43]. While sheep and horse blood is often not readily available in many developing countries, goat or pig blood is more accessible and affordable and could be used for agar production [44]. A further reason for abandoning the use of human blood agar is the associated biohazard to the laboratory personnel due to frequent infection with HIV, hepatitis B and C among blood donors.

## Conclusion

Our study shows that antimicrobial resistance predicts fatal outcome of bloodstream infection. While malaria can be treated with highly effective, inexpensive and widely available quinine, treatment of bacterial and fungal bloodstream infection is compromised by antimicrobial resistance and unavailability of expensive second-line antibacterial and systemic antifungal drugs. The finding that antimicrobial resistance, HIV-infection and malnutrition independently predicts fatal outcome not only call for renewed efforts to improve HIV care, nutrition and general living conditions for children, but also argue for increased focus on interventions to limit the selection pressure for resistant organisms such as education on rational use of antimicrobials, development of judicious guidelines for treatment strategies and adequate government regulation of drug quality and access to antimicrobial drugs [45].

## Competing interests

The author(s) declare that they have no competing interests.

## Authors' contributions

BB, KPM, WKU, BST, DSM, RJ, VM, MGT, SH, SYM and NL conceived and designed the study. The study clinicians were KPM, BST and other medical doctors in the Department of Pediatrics and Child Health. VM, MGT, BB and RJ analyzed the cultures. BB and MGT did the HIV rapid tests and MHP performed the HIV PCR. BB did the statistical analyses with contributions from NL, SH and other authors. All authors contributed to the interpretation of the results and writing of the manuscript.

## Pre-publication history

The pre-publication history for this paper can be accessed here:


